# Gastric Ulcer

**Published:** 1878-07

**Authors:** 


Gastric Ulcer.—Through the kindness of Dr. J. M. Stevenson, I was present at the *post-mortem* examination, and I am indebted to him for the notes of the following case:

Mrs. J. B., aged 55, widow; mother of six children; always healthy, except habitually constipated. Was called to see her on February 12, 1877. Suffering pain in stomach and flatulence after eating. Had several times vomited a thin, whitish water, which did not smell sour nor appear gluey or ropy. General health not affected.

Prescribed for functional disease of stomach consequent on constipation.

February 19.—Reports herself relieved of all pain and vomiting, and feels well. Prescribed a glass of Carlsbad water every morning before breakfast, and care in diet.

July 30.—Had a slight chill and now some fever. Has had several loose stools, and has voided mucus stained with blood, attended with griping pains and tenesmus. Prescribed for slight dysentery: magnesiæ sulph., acidi sulph. aromatici et tr. opii.

August 3.—Reports herself as feeling entirely well.

January 24, 1878.—Reported through a neighbor that she was again vomiting whitish water. Prescribed bismuth, pepsin and opium.

January 26.—Reports no relief. On calling to see her, she reports her health as having been fairly good since the attack of dysentery. A good appetite, partaking of the same food as the rest of the family, except fruits and sour things, which swelled her stomach and caused pain; had had no return of vomiting or pyrosis until the first of January. Within the past two weeks could take no solid food, on account of it causing pain. She appears as well nourished as in July, and has no cachexia to indicate organic disease of any nature. Tenderness on pressure over epigastrium. Bowels have not moved for four days. Suffers from thirst. Tongue heavily coated. Prescribed creasote, hydrocyanic acid, and morphia, and ordered enema of castor oil and turpentine.

January 27, 9 A.M.—Vomiting ceased since taking first dose of medicine; but bowels have not moved, although irritating enema was followed by a quart of soapsuds. Enema did not come away. Ordered eight ounces of English black draught, one-third to be taken every four hours, unless bowels moved.

January 27, evening.—Not so well. Enema retained. Abdomen slightly tympanitic. Thirst and nausea, but no vomiting. Expression anxious. Tongue heavily coated with a dirty-yellow fur. Pulse 90; temperature 100½°. Suspected obstruction of the bowel. Ordered hot linseed poultice to abdomen, and a pill of morph. sulph. et ext. belladonna aa gr. ¼, every four hours.

January 28.—Expresses herself as better; free from pain. Had slept several hours. General appearance the same. Tympanitis increased, and more general tenderness over abdomen. Introduced rectum tube, through which the enema slowly flowed away. Carried the tube up about twenty inches, when it was arrested; through it, by means of a siphon syringe, injected five pints of warm water, when it began to flow out by side of the tube. Continued pills and poultice.

January 28, evening.—Condition much the same. Enema has not come away. Treatment continued.

January 29, morning.—She feels better; slept four hours at a time. Pulse 90; temp. 100°. Abdominal distention slightly increased. Tenderness only slight. Great thirst. Bowels not moved. Evening—no change.

January 30, morning.—Feels better; thinks she voided some flatus. Symptoms all seem more favorable, and indicate a yielding of the obstruction.

January 30, evening.—Patient moribund. They report that about 10 A.M. she asked to be raised up in bed, and drank some mutton broth, when she suddenly became faint and went into a collapse. Died at 8 P.M.

*Autopsy*, 19 hours after death.—Body moderately emaciated; rigidity slight. Abdomen prominent and tympanitic. On section, evident peritonitis, but slight and recent. Lymph effused, and gluing more or less tightly intestines and abdominal contents. The small and large intestines to the termination of the transverse colon fully distended with gas, and from this point the descending colon and rectum were collapsed. The obstruction was apparently bands of lymph constricting walls of colon, but hardly sufficient to have caused permanent constriction, as on pressure the gas in the upper intestine was forced through. On raising left lobe of liver, a mass of straw-colored lymph was seen, and in the middle of the lesser curvature of the stomach was noticed an oval opening about four lines in diameter, the peritoneal covering having evidently been destroyed some time, and the contents of the stomach having been prevented from escaping by its adhesions to the left lobe of the liver. On opening the stomach, an elliptical ulcer, about an inch and three-fourths in its long diameter, was found on the lesser curvature, corresponding to external opening observed. The walls of the ulcer were three-quarters of an inch thick, and the adjacent coats of the stomach infiltrated with cellular deposit. The stomach opposite the ulcer was slightly congested, and the whole calibre of the organ apparently diminished in size.

This case is markedly interesting from its negative history, having never arrested especially the attention of the patient nor presented any symptoms from which a diagnosis could have been definitely determined. Again, it is interesting from the fact that the adhesions for a time prevented death, and at last caused it. Those forming a new floor prevented extravasation; but those gluing the coils of intestines together, and obstructing their functions, 'caused accumulation of gas and distention of transverse colon and upper intestine, which, by alteration of the relation of the parts, caused the new adhesions of the stomach and liver to give way on the patient being raised up.—*Philapelphia Medical Times.*

---

Hints on the Hypodermic use of Morphia.—A death occurred not long since in Washington City from the hypodermic injection of the third of a grain of sulphate of morphia, in the case of an adult laboring under pneumonia. A distressing cough had led to the administration of several doses of Dover's powder during the day, which failing to give relief, the morphia was introduced in the evening. Narcosis speedily ensued, and in spite of well-judged antidotal efforts, death followed in a few hours. The patient had frequently used opium and morphia with no bad effect.

This is not the only instance that has come to my knowledge of death attributed to the hypodermic injection of morphia. Some years ago a prominent individual in one of our California cities lost his life under similar circumstances; and the event threw so much discredit on the hypodermic use of morphia, that the practice has been almost abandoned in that locality. I have heard

